# Effect of heart rate reduction with ivabradine on quality of life in advanced cancer patients

**DOI:** 10.1093/eschf/xvag051

**Published:** 2026-03-31

**Authors:** Yvonne Bewarder, Angela Zimmer, Markus Anker, Stefan Anker, Konstantinos Christofyllakis, Moritz Bewarder, Igor Schwantke, Michael Böhm

**Affiliations:** Universitätsklinikum des Saarlandes, Klinik für Kardiologie, Angiologie und internistische Intensivtherapie, Saarland University, Kirrberger Str. 1, Homburg 66421, Germany; Universitätsklinikum des Saarlandes, Klinik für Kardiologie, Angiologie und internistische Intensivtherapie, Saarland University, Kirrberger Str. 1, Homburg 66421, Germany; Department of Cardiology, Angiology and Intensive Care Medicine CBF, German Heart Center Charité, Berlin, Germany; Charité University Medicine Berlin Corporate Member of Free University Berlin and Humboldt-University Berlin, Berlin, Germany; German Centre for Cardiovascular Research Partner Site Berlin, Berlin, Germany; School of Cardiovascular and Metabolic Health, University of Glasgow, Glasgow, UK; Department of Cardiology (CVK) of German Heart Center Charité, German Centre for Cardiovascular Research (DZHK) Partner Site Berlin, Charité Universitätsmedizin, Berlin, Germany; Universitätsklinikum des Saarlandes, Klinik für Haematologie und Onkologie, Saarland University, Homburg, Germany; Universitätsklinikum des Saarlandes, Klinik für Haematologie und Onkologie, Saarland University, Homburg, Germany; Centrum für Hämatologie und Onkologie Bethanien, Frankfurt am Main, Germany; Universitätsklinikum des Saarlandes, Klinik für Kardiologie, Angiologie und internistische Intensivtherapie, Saarland University, Kirrberger Str. 1, Homburg 66421, Germany; Universitätsklinikum des Saarlandes, Klinik für Kardiologie, Angiologie und internistische Intensivtherapie, Saarland University, Kirrberger Str. 1, Homburg 66421, Germany; HOMICAREM (HOMburg Institute for CArdioREnalMetabolic Medicine), Universitätsklinikum des Saarlandes, Saarland University, Homburg, Germany

**Keywords:** Ivabradine, Advanced cancer, quality of life

Heart failure (HF) and cancer share mutual mechanistic pathways^1^ and both are characterized by increasing symptoms leading to severe clinical impairments and reduced self-care.^2^ Heart rate (HR) is elevated in patients with cancer^3^ and HF,^4^ where it is associated with outcomes^3,4^ and quality of life (QoL),^5^ in particular when blood pressure is low.^6^ Ivabradine reduces HR^4,5^ and associates with an improvement of QoL^5^ and outcomes across the risk spectrum in HF.^4^ Recently, the EMPATICC trial has shown in a multi-pharma intervention with HF drugs, some changes in QoL in cancer patients.^7,8^ In EMPATICC, a multi-drug combination therapy was used with ivabradine, sacubitril/valsartan, SGLT2-inhibitors and iron therapy. In this paper we only wanted to look at those patients that received ivabradine exclusively to better understand the effects this medication alone could have on quality of life and clinical parameters.^7^

We report on our clinical experience of using ivabradine to lower HR in patients with cancer presenting with high HR and complaining about symptoms of shortness of breath. Ethical approval was obtained from the Ethics Committee of the Medical Association of Saarland (Ärztekammer des Saarlandes) for both the Heart Failure and Homburger Cardio-Oncology registries. Between May 2019 and October 2022, we included 12 patients with various types of advanced cancer without the history of HF, a HR ≥ 70 bpm and severe impairment of QoL. All patients underwent chemotherapy or immune-therapy. As there are no comparable, validated tests for quality of life assessment for cardiological patients without HF, QoL was assessed using Kansas City Cardiomyopathy Questionnaire (KCCQ) and the Minnesota Living with Heart Failure Questionnaire (MLHFQ). Pre- and post-ivabradine values were compared using the Wilcoxon signed-rank test for paired samples. Results are presented as median (Inter Quartile Ranges, IQR) with an alpha of 5% as well as using the paired Student’s *t*-test (results are presented as mean (±SEM) with an alpha of 5%).

Resting HR was high (mean 96.4 bpm) and systolic blood pressure (SBP) low (mean 107.5 mmHg). There was an elevation of inflammatory markers such as CRP, without evidence of an acute infection with a normal body temperature (mean 36.7°C). Patients with HF were excluded. For arterial hypertension, 58.3% of patients were on beta-blockers, 50% on ACE inhibitors and 58.3% on diuretics. The ejection fraction was normal or mildly reduced. Ivabradine reduced HR by median change of 25.6 bpm (*P* < .001) without effecting blood pressure (data not shown). Ivabradine therapy was associated with significant improvement of KCCQ from baseline concerning symptom frequency, total symptom score and symptom stability, while other KCCQ domains showed numerical improvements. There was no heterogeneity between the different components of KCCQ (*Figure 1A* and *B*). MLHFQ showed no significant changes (data not shown).

**Figure 1:**
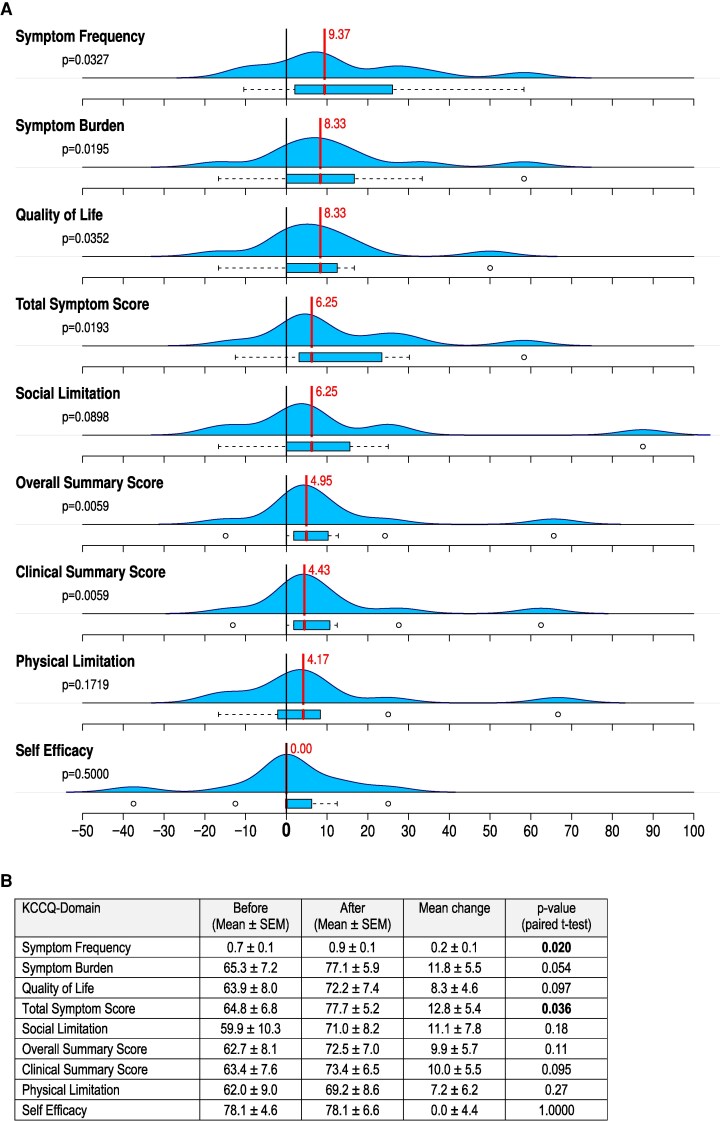
(*A*) Box plots and density plots showing Kansas City Cardiomyopathy Questionnaire (KCCQ) domain changes from baseline to post-treatment of patients with ivabradine. (*B*) Kansas City Cardiomyopathy Questionnaire (KCCQ) domain scores before and after ivabradine (4–6 weeks after start) therapy. Mean change and *P*-values (paired *t*-test) are presented

Resting HR associates with poor outcomes in HF,^4^ in particular when blood pressure is low^6^ and has significant effects on QoL^5^ and ivabradine improves symptoms and associates with a reduction of outcomes^4^ in HF.^5^ This observation shows that HR reduction with ivabradine in cancer patients, in whom HR is usually elevated,^3^ improves QoL. The limitation is that this represents a report on a limited number of patients without a control group. However, the observations were striking and novel. This investigation should set the stage to follow the concept of the EMPATICC trial,^7^ which provided the first step to improve QoL symptoms and self-care in cancer patients. This observation argues that there could be interactions between the single components of such combination treatment and that the concept of HR reduction should be prospectively tested in the condition of severe cancer or even other chronic conditions^8^ in a randomized controlled fashion.

## Declarations

### Data Availability

The data underlying this study are derived from the Homburg Cardio-Oncology and Heart Failure registries. Access to de-identified data can be granted by the corresponding author upon reasonable request.
